# Specific targeting of the deubiquitinase and E3 ligase families with engineered ubiquitin variants

**DOI:** 10.1002/btm2.10044

**Published:** 2016-11-14

**Authors:** Maryna Gorelik, Sachdev S. Sidhu

**Affiliations:** ^1^ Banting and Best Dept. of Medical Research and the Dept. of Molecular Genetics Terrence Donnelly Center for Cellular and Biomolecular Research, University of Toronto 160 College Street Toronto ON Canada M5S 3E1.

**Keywords:** APC/C, DUBs, phage display, protein engineering, SCF E3 ligases, small protein scaffolds

## Abstract

The ubiquitin proteasome system (UPS) has garnered much attention due to its potential for the development of therapeutics. Following a successful clinical application of general proteasome inhibitors much effort has been devoted to targeting individual UPS components including E3 enzymes and deubiquitinases that control specificity of ubiquitination. Our group has developed a novel approach for targeting the UPS proteins using engineered ubiquitin variants (Ubvs). These drug‐like proteins can serve as valuable tools to study biological function of UPS components and assist in the development of small molecules for clinical use. In this review, we summarize studies of Ubvs targeting members of three major families, including deubiquitinases, HECT E3 ligases, and CRL E3 ligases. In particular, we focus on Ubv binding mechanisms, structural studies, and effects on enzyme function. Furthermore, new insights gained from the Ubvs are discussed in the context of small molecule studies.

## 1 Introduction

Ubiquitination plays a central role in controlling the stability and function of cellular proteins. Approximately a thousand of genes in the ubiquitin (Ub) proteasome system (UPS) are involved in controlling ubiquitination in a specific and timely manner by marking proteins for degradation by the proteasome or by regulating their function. The misregulation of UPS proteins has been increasingly linked to human diseases, in particular cancer, and as a result the UPS is considered important for therapeutic development. Unlike kinases, which have been targeted by numerous small molecule drugs,^1^ development of small molecules targeting UPS components has lagged behind. To date, only general proteasome inhibitors^2^ and thalidomide derivatives acting on CRBN^3^ have been approved for treatment of haematologic malignancies. Additionally, a few compounds targeting specific UPS components^4^, ^5^ or families^6^ are in clinical trials. However, the UPS contains hundreds of proteins and the therapeutic potential of targeting specific components remains largely unexplored.

**Figure 1:**
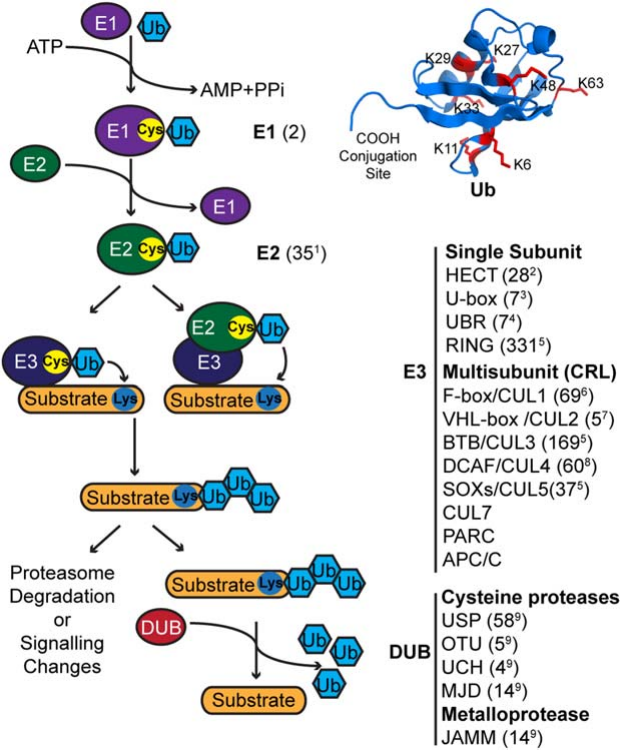
The ubiquitin proteasome system. The cartoon shows an overview of the ubiquitination process and the major components of the UPS. In the first step of Ub conjugation, E1 consumes ATP to form a high energy thioester bond between the Ub C‐terminal carboxyl group and the E1 active site cysteine thiol group. In the next step, the activated Ub is transferred to the E2 active site cysteine. In the final step, E3 mediates conjugation of the Ub C‐terminal carboxyl group to an amino group of a lysine residue in a substrate protein or another Ub molecule. In this step, the Ub is either transferred through an E3 active site cysteine (HECT E3 ligases) or directly from an E2 to the receiving lysine. DUBs catalyze the cleavage of the Ub C‐terminal carboxyl from substrate proteins or from Ub chains to reverse ubiquitination. The structure of Ub (PDB: 1UBQ) is shown (top right), indicating the location of the C‐terminal carboxyl conjugation site and the seven acceptor lysine residues, which are shown as red sticks. The number of members in each UPS family is indicated in parenthesis and was taken from the following studies: 1,^7^ 2,^8^ 3,^9^ 4,^10^ 5,^11^ 6,^12^ 7,^13^ 8,^14^ 9^15^

The central player of the UPS is Ub, a post‐translational protein modifier that is highly conserved across eukaryotes. Ub conjugation is accomplished through the sequential actions of ubiquitin‐activating (E1), ubiquitin‐conjugating (E2), and ubiquitin‐ligating (E3) enzymes that are responsible for binding substrates and regulating specificity of ubiquitination (Figure 1). The central role of the E3 ligases is reflected in a large number of ∼600 E3 ligases encoded in the human genome, compared with only 35 E2 enzymes and two E1 enzymes. The E3 ligases can act either as catalytic intermediates, represented by Homologous to E6‐AP Carboxy Terminus (HECT)‐type E3 ligases in humans, or as adaptor proteins that mediate the transfer of Ub directly from E2 conjugating enzymes to substrates (Figure 1). The majority of the human E3 ligases belong to the latter class, and share the Really Interesting New Gene (RING) domain that recruits E2 enzymes. The RING E3s are further subdivided into single subunit families and multisubunit Cullin RING E3 ligase (CRL) superfamily (Figure 1). While enzymes of the E1, E2, and E3 families mediate ubiquitination, the family of ∼100 deubiquitinating enzymes (DUBs) carry out the reverse process of cleaving Ub moieties (Figure 1). Substrate proteins can be either monoubiquitinated or polyubiquitinated, where the topology of the polyubiquitin chains is determined by which of the seven Ub lysines is used for cross‐linking (Figure 1). Typically, K48‐ and K11‐linked chains mark proteins for degradation by the 26S proteasome, while other type of linkages alter the localization and/or activity of modified proteins. The latter is accomplished by proteins containing Ub binding domains (UBDs) that constitute another important class of UPS proteins that act as readers of Ub modifications.

Therapeutic targeting of UPS components can benefit greatly from the use of intracellular drug‐like protein molecules, which are easier to generate than small molecule compounds, but like small molecules can be used to explore biological outcomes of targeting a specific protein site. Previous work demonstrated that Ub is amenable to genetic engineering, where de novo binders to cell‐surface receptors were generated through ribosome display by varying several positions on the Ub surface.^16^, ^17^ However, engineered Ub variants (Ubvs) are particularly suited for targeting components of the UPS.^18^ This is because virtually all UPS proteins already contain weak Ub‐binding sites including active and regulatory sites.^19^, ^20^, ^21^ While targeting active sites with high affinity Ubvs is expected to antagonize function, targeting regulatory sites may have a spectrum of outcomes ranging from antagonistic to agonistic.

Here we summarize the results and insights gained through targeting of three major UPS families with Ubvs. We describe the generation of Ubv inhibitors targeting DUBs, which was the first demonstration of the technology and laid the foundation for following studies. Next, we describe Ubvs generated against a family of HECT E3 ligases, which provided the first example of Ubvs acting as activators. Finally, we describe the development of Ubvs against CRLs including the SKP1‐CUL1‐F‐box (SCF) family and the Anaphase Promoting Complex/Cyclosome (APC/C) complex.

## 2 Inhibitors of DUB proteases

The human genome encodes 116 DUBs that are subdivided into five families based on the structures of their catalytic domains, including four families of cysteine proteases and a metalloprotease family^15^ (Figure 1). DUBs constitute an important class of therapeutic targets with numerous family members implicated in a variety of diseases including cancer and neurodegenerative, infectious, and blood diseases.^22^ Despite extensive efforts, potent small molecules have only been developed against a small number of DUBs, and most of these inhibitors exhibit low specificity and potency.^23^ Furthermore, there are no published structures of human DUBs in complex with small molecule inhibitors, and this has prevented detailed understanding of the inhibition mechanisms to guide further design.

**Figure 2:**
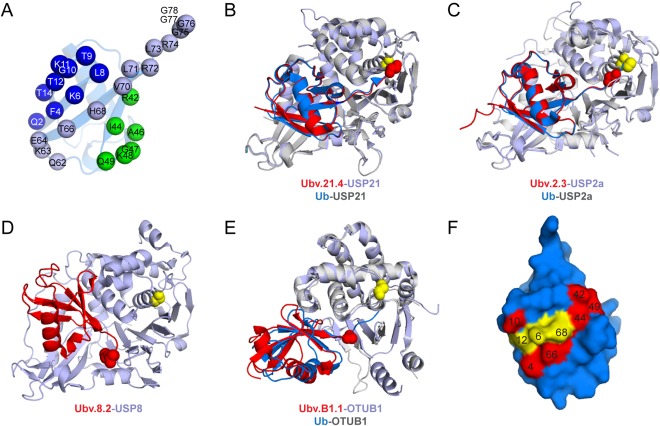
Ubv inhibitors of DUBs. (A) The Ub library designed to randomize surface residues involved in interactions with USP DUBs.^18^ Residues randomized in the library are indicated as spheres on the structure of Ub (PDB: 1UBQ) and colored according to the targeted region: Region 1, *dark blue*; Region 2, *green*; Region 3, *light blue*. (B), (C), (D), and (E) Structures of Ubvs in complex with DUBs: Ubv.21.4‐USP21 (PDB: 3MTN) (B), Ubv.2.3‐USP2 (PDB: 3V6E) (C), Ubv.8.2‐USP8 (PDB: 3N3K) (D), Ubv.B1.1‐OTUB1 (PDB: 4I6L) (E). Ubvs and DUBs are colored *red* or *light blue*, respectively. Catalytic cysteine residues of DUBs are shown as *yellow* spheres and the C‐ termini of Ubvs are shown as *red spheres*. Ubv.21.4‐USP21 (B), Ubv.2.3‐USP2(C), and OTUB1‐Ubv.B1.1 (E) complexes are shown in superposition with Ub.wt‐USP21 (PDB: 3I3T), Ub.wt‐USP2 (PDB: 2HD5), or Ub.wt‐OTUB1 (PDB: 4DDG), respectively, which are colored as follows: Ub, *blue*; DUB, *grey*. (F) The core functional epitope identified by saturation scanning mutagenesis analysis of Ubv.2.3 and Ubv21.4 interactions with USP2 and USP21, respectively.^25^ The Ub is shown as surface and the residues of the identified core functional epitope are labeled and colored *red* or *yellow* if they were the same or different, respectively, in Ubv.2 and Ubv.21

Motivated by the paucity of effective inhibitors of DUBs, our group developed an approach to generate potent and specific Ubvs as protein‐based inhibitors.^18^ The Ub specific protease (USP) subfamily contains a conserved binding site for the distal Ub that is conjugated through its C‐terminal moiety to lysine in other Ub or protein substrates. Based on analysis of available USP‐Ub structures, we targeted for combinatorial mutagenesis approximately 30 residues on Ub that interact with the USP Ub‐binding site (Figure 2A). Resulting libraries containing billions of Ubvs were displayed on phage and subjected to binding selections for particular DUBs and other Ub‐associated proteins. This strategy was successful in generating tight and specific Ubvs binding to USP2, USP8, or USP21 (Table 1). Additionally, this approach also generated specific Ubvs for the: DUBs OTUB1 and JAB1, members of the ovarian tumor protease (OTU) and JAB1/MPN/MOV34 (JAMM) subfamilies, respectively; E2 conjugating enzyme Cdc34; HECT E3 ligases NEDD4 and ITCH; and the non‐catalytic UBD of USP37. These results showed that the common Ub epitope that engages many Ub‐associated proteins can be fine‐tuned for specific targeting of particular proteins.

**Table 1:** Characteristics of Ubv inhibitors of DUBs

Ubv (target)	Mutations^a^	Affinity^b^	Biological activity	PDB code
Ubv.2.3 (USP2)	3(3)	25 nM		3V6E
Ubv.8.2 (USP8)	12(9)	4.8 nM	Inhibits EGFR deubiquitination and decreases EGFR levels	3N3K
Ubv.21.4 (USP21)	3(3)	2.4 nM	Inhibits RIP1 deubiquitination and enhances NF‐κB activation	3MTN
Ubv.B1.1 (OTUB1)	6(4)	20 nM		4I6L

a The number of amino acid substitutions in Ubv relative to Ub.wt. The number of substitutions that are located within the binding interface is indicated in parenthesis.

b Concentration of Ubv that decreased USP activity by 50% (IC_50_) is reported for USP2, USP8, and USP21, and the dissociation constant measured by time resolved Forster‐energy transfer (TR‐FRET) is shown for OTUB1.^18^

The structures of USP2, USP21, USP8, and OTUB1 complexes revealed that the Ubvs bound to the distal Ub‐binding site and acted as inhibitors of diUb cleavage. However, the Ubv interaction mode varied between different complexes. In the USP2‐Ubv.2.3 and USP21‐Ubv.21.4 complexes, the Ubv bound in an orientation that was virtually identical to that of wild‐type Ub (Ub.wt) (Figure 2B,C). Notably, these Ubvs contained only three substitutions relative to the Ub.wt, demonstrating that strengthening just a few key interactions can lead to dramatic improvements in affinity and specificity (Table 1). In the case of OTUB1, although the Ubv bound in a similar orientation, it was slightly rotated relative to Ub.wt (Figure 2E). However, still only a small number of substitutions relative to Ub.wt were sufficient to generate high affinity and specificity (Table 1). Ub.wt is known to allosterically activate the binding of OTUB1 to UbcH5b‐Ub complex^26^ and the Ubv also re‐capitulated this property, albeit less efficiently despite much higher affinity. This highlights the fine‐tuned nature of Ub‐substrate interactions, where even small changes in binding mechanism can lead to altered function. Finally, in the case of USP8, there is no structure available for the complex with Ub.wt, but the Ubv contained many mutations and bound in an orientation that was drastically different from that expected for an Ub substrate, as the tail of Ubv.8.2 pointed away from the active site cleft (Figure 2D) (Table 1). Ubv.8.2 also displayed the highest specificity, binding only USP8, whereas both Ubv.2.3 and Ubv.21.4 showed weak binding to other UPS proteins.^18^ Thus, many substitutions in Ubvs can work together to produce drastic changes in the binding mode, resulting in high specificity and affinity. Ubvs targeting USP8 and USP21 were validated in cellular assays and, consistent with high affinity and specificity, they co‐immunoprecipitated with their cognate USPs, blocked ubiquitination of endogenous protein substrates, and modulated the activity of the pathways regulated by the USPs (Table 1).

The high affinities of Ubvs generated against DUBs and other UPS proteins make them valuable tools to explore functional details of Ub interaction, which is otherwise difficult to investigate due to the low affinity and promiscuity native interactions. For example, we took advantage of the virtually identical binding modes between Ub.wt and Ubv.2.3 or Ubv.21.4 to investigate the molecular details of Ubv‐USP interactions.^25^ Strikingly, saturation scanning mutagenesis of Ubvs revealed a contiguous nine‐residue epitope that was conserved for both Ubv.2.3 and Ubv.21.4 binding (Figure 2F). Notably, six of nine residues in the core functional epitope were conserved as the wt in Ubv.2 and Ubv.21, and these were involved in conserved interactions between the Ubvs and USPs. In contrast, the three residues that differed in Ubv.2 or Ubv.21 relative to Ub.wt clustered together and mediated different interactions with USPs that could be exploited to generate high specificity inhibitors (Figure 2F). Similar analyses could be extended to dissect other Ub interactions with diverse members of the UPS and should prove useful for structure‐guided design of specific inhibitors.

Others have also generated Ubv inhibitors of other USPs.^27^, ^28^ In contrast to our approach which targeted surface exposed Ub residues involved in USP binding, these studies randomized computationally selected residues predicted to affect the conformation of the β1‐β2 loop that interacts with USPs. Phage display was used to generate Ubvs with submicromolar affinities for USP14 and reduced affinities for the UCH DUB subfamily.^27^ NMR analysis demonstrated that substitutions in these Ubvs do not cause detectable changes in the β1‐β2 loop conformational state, but rather, slow down its conformational motions, which highlights the importance of conformational dynamics for Ub interactions. The Ubv library designed to alter the β1‐β2 loop conformation was also used to generate binders to USP7 and affinity was further improved with additional surface mutations.^28^ The Ubv inhibited catalytic activity, but the structure of the Ubv‐USP7 complex was not solved, preventing characterization of the binding mechanism. These results further highlight the amenability of the Ub scaffold for generation of Ubvs targeting the DUB family.

In summary, tight and specific Ubv inhibitors have been generated against several DUBs and have been shown to be useful tools for exploring molecular details of DUB interactions and for investigating biological consequences of inhibition. While all structurally characterized Ubvs bound to the distal Ub‐binding site and blocked substrate binding, it is intriguing to speculate that other Ub‐binding sites on DUBs^29^ may be targeted to generate modulators that alter rather than block function. The same may be true for members of other UPS families and, as described below, additional studies have focused on HECT and CRL families of E3 ligases with the goal of generating Ubvs against known and previously uncharacterized Ub binding sites.

## 3 Modulators of HECT E3 ligases

**Figure 3:**
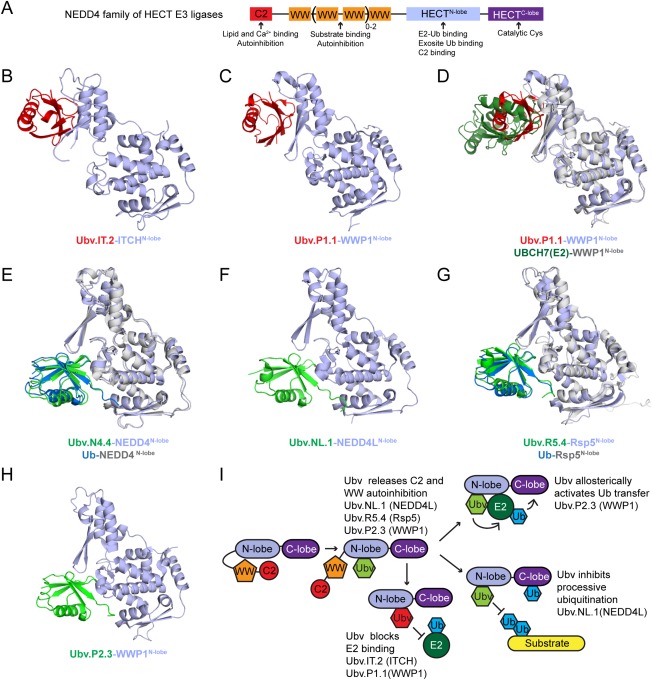
Ubv inhibitors, activators, and modulators of HECT E3 ligases. (A) Domain composition of the NEDD4 subfamily of HECT E3 ligases. (B) and (C) Structures of Ubv inhibitor complexes: Ubv.IT2‐ITCH (PDB: 5C7M) (B) and Ubv.P1.1‐WWP1 (PDB: 5HPS) (C). Only the N‐lobe of HECT domains is shown. Ubv and HECT N‐lobe are colored *red and light blue*, respectively. (D) Superposition of Ubv.P1.1‐WWP1 and UBCH7‐WWP1 (PDB: 5HPT) complexes showing that Ubv inhibitors target the E2 binding surface. Structural alignment was produced by aligning WWP1 HECT N‐lobe domains. The Ubv.P1.1‐WWP1 complex subunits are colored as in (B) and the UBCH7‐WWP1 complex subunits are colored as follows: WWP1 N‐lobe, *grey*; UBCH7, *dark green*. (E), (F), (G), and (H) Structures of Ubv activator/modulator complexes: Ubv.N4.4‐NEDD4 (PDB: 5CJ7) (E), Ubv.NL1.1‐NEDD4L (PDB: 5HPK) (F), Ubv.R5.4‐Rsp5 (PDB: 5HPL) (G), and Ubv.P2.3‐WWP1 (PDB: 5HPT) (H). Only the N‐lobe of HECT domains is shown. Ubv and HECT N‐lobe are colored *green* and *light blue*, respectively. Ubv.N4.4‐NEDD4 (E) and Ubv.R5.4‐Rsp5 (F) complexes are shown superimposed with Ub.wt‐NEDD4 (PDB: 4BBN) or Ub.wt‐Rsp5 (PDB: 3OLM), respectively. Structural alignment was produced by aligning N‐lobe of HECT domains. Subunits of Ub‐NEDD4 and Ub‐Rsp5 complexes are colored as follows: Ub.wt, *blue*; HECT N‐lobe, *grey*. (I) Schematic of HECT3 ligase activation and catalysis depicting observed roles of different Ubvs

Of the ∼600 E3 ligases encoded by the human genome, 28 belong to the extensively characterized HECT family, and these contribute to many essential cell processes and have been linked to numerous diseases.^30^ HECT family members share a conserved C‐terminal catalytic HECT domain that is composed of the flexibly tethered N‐lobe and C‐lobe. The N‐lobe binds to an E2 enzyme charged with Ub, while the C‐lobe receives the Ub transferred from the E2 enzyme (Figure 3A). In addition to the catalytic HECT domain, HECT E3 ligases possess various N‐terminal sequences with a variety of domains that are involved in substrate binding and regulation of ligase activity. Members of the NEDD4 HECT subfamily share an architecture that is conserved across all eukaryotes and consists of an N‐terminal C2 domain, followed by several WW domains and the C‐terminal HECT domain (Figure 3A). Many NEDD4 family members were shown to be regulated by an autoinhibitory interaction between the HECT domain and either the C2 domain in the case of SMURF2 and NEDD4,^31^, ^32^ the WW domains in the case of ITCH,^33^ or both in the case of WWP1 and WWP2.^34^, ^35^ In addition to the C‐lobe active site that interacts with Ub, members of the NEDD4 family also contain a weak Ub‐binding N‐lobe exosite.^36^ Several studies suggested that binding of the substrate‐linked Ub to the N‐lobe exosite enhances polyubiquitination by orienting the distal end of growing polyubiquitin chains^37^, ^38^, ^39^ and by enhancing the processive ubiquitination mode.^40^ Additionally, the N‐lobe exosite was shown to directly overlap with the C2 binding surface on SMURF2 and NEDD4, suggesting that Ub binding to this site might also contribute to relieving C2 mediated autoinhibition.^41^

To date only a handful of small molecules targeting HECT E3 ligases have been reported, including a general HECT inhibitor^42^ and an ITCH inhibitor that target active sites,^43^ a SMURF inhibitor that disrupts substrate binding,^44^ and a NEDD4 inhibitor that binds the N‐lobe exosite and inhibits processive ubiquitination.^40^ While these small molecules provide some insight into different mechanisms that can modulate HECT function, the small number of available molecules, lack of structural information (a structure is only available for the NEDD4 inhibitor), and lack of potency and specificity in some cases suggest that alternative methods are required to systematically investigate the mechanisms and biological outcomes of specifically targeting HECT E3 ligases across the whole family.

We used the phage‐displayed Ubv library (Figure 2A) to target HECT domains from 19 human proteins (including all members of the NEDD4 family) and from Rsp5, the yeast homologue of human NEDD4.^18^, ^45^ These selections yielded a total of 69 unique Ubvs, which displayed high affinity and specificity for their targets, although some cross‐reactivity was observed for closely related homologs (Table 2). The Ubvs acted as inhibitors, activators or modulators of ubiquitination levels in autoubiquitination assays with their targets^45^ (Table 2). To investigate mechanisms behind inhibition, activation and modulation, the structures of six distinct HECT‐Ubv complexes were solved.

**Table 2:** Characteristics of Ubv modulators of HECT E3 ligases

Ubv (target)	Mutations^a^	Affinity^b^	Specificity^c^	Activity (binding site)	Biological activity^d^	PDB
Uv.IT.2 (ITCH)	15(9)	12 µM	Specific	Inhibitor (E2 site)		5C7M
Ubv.P1.1 (WWP1)	17(9)	320 nM	WWP2 (1.6 µM)	Inhibitor (E2 site)		5HPS
Ubv.NL.3 (NEDD4L)	5	840 nM	Specific	Inhibitor	Increases levels and function of NEDD4L substrate ENaC.	
Ubv.HA3.1 (HACE1)	13	580 nM	Specific	Inhibitor	Inhibits ubiquitination of HACE1 substrate Rac1 Activates cell migration	
Ubv.HU.1 (HUWE1)	14	120 nM	Specific	Inhibitor	Stabilizes HUWE1 and its substrate c‐Myc	
Ubv.S2.5 (SMURF2)	16	470 nM	Specific	Inhibitor	Inhibits cell migration.	
Ubv.N4.2 (NEDD4)	13	4.2 µM	Nedd4L (9.3 µM)	Inhibitor	Increases polyubiquitination of NEDD4 substrate YY1	
Ubv.N4.4 (NEDD4)	8(5)	93 µM	Nedd4L (53 µM)	Activator (N‐lobe exosite)		5C7J
Ubv.P2.3 (WWP2)	12(5)	78 nM	WWP1 (230 nM)	Activator (N‐lobe exosite)	Promotes polyubiquitination of WWP2 and reduces protein levels of WWP2 and its substrate PTEN. Inhibits cell migration.	5HPT
Ubv.NL.1 (NEDD4L)	14(10)	9.7 nM	NEDD4 (210 nM)	Modulator (N‐lobe exosite)	Decreases levels and function of characterized NEDD4L substrate ENaC and new substrate RhoB. Inhibits cell migration.	5HPK
Ubv.R5.4 (yRsp5)	8(4)	130 nM	Not determined	Modulator (N‐lobe exosite)		5HPL

a The number of amino acid substitutions in Ubv relative to Ub.wt is shown. For Ubv with solved complex structures the number of mutated residues that are located within the Ubv binding interface is indicated in parenthesis.

b Dissociation constants were measured using bio‐layer interferometry (BLI) by immobilizing HECT domains on the biosensor and measuring binding of Ubvs.^45^

c Specificity was determined by assessing binding to a panel of 20 different HECT domains. For Ubvs with observed cross‐reactivity, the cross‐reactive HECT domains and associated affinities are indicated.^56^

d Function in cells as determined for Ubv.N4.2 by^18^ and all other Ubvs by^45^. Effect on the cell migration of HCT116 cells is indicated for Ubvs shown to influence cell migration in a lentiviral screen with pooled Ubvs.^45^

The structures of ITCH and WWP1 in complex with their Ubv inhibitors (Ubv.IT.2‐ITCH and Ubv.P1.1‐WWP1) revealed that these Ubvs targeted the E2 binding site, rather than the active site as observed for small molecule inhibitors (Figure 3B–D). Functional assays further confirmed that these Ubvs blocked the transfer of Ub from E2 to E3, presumably by preventing E2 binding. Strikingly, Ubv.IT2 and Ubv.P1.1 bound in a very similar orientation (Figure 3B,C), raising the possibility that these Ubvs target a previously uncharacterized natural Ub binding site that is involved in regulating some aspect of HECT E3 ligase function. Cyclic peptide inhibitors were also generated against the E2 binding sites of NEDD4, WWP1, SMURF2, and HUWE1 HECT E3 ligases^42^ and, combined with our results, validate this surface as a promising site for inhibition of HECT E3 ligases.

The structures of WWP1, NEDD4, NEDD4L, and Rsp5 in complex with their Ubv activators (Ubv.N4.4‐NEDD4, Ubv.P2.3‐WWP1) or modulators (Ubv.NL.1‐NEDD4L, Ubv.R5.4‐Rsp5) revealed that in these cases the Ubv bound to the N‐lobe exosite. Furthermore, all four Ubvs bound in a similar orientation, which was similar to the orientation of Ub.wt in complex with NEDD4 or Rsp5 (Figure 3E–H). Thus, as observed in the case of the Ubvs targeting USP2, USP21, and OTUB1, these Ubvs have acquired substitutions that strengthen key interactions with targets while preserving the Ub.wt binding mode.

In vitro assays with WWP1, NEDD4L, and Rsp5 revealed that, despite a common binding mechanism, Ubvs regulate the function of these ligases in different ways. Assays that monitored transfer of a single Ub demonstrated that all Ubvs activated this aspect of the ubiquitination reaction, while the presence of both C2 and WW domains exerted an inhibitory effect. In NEDD4L and Rsp5, removal of both C2 and WW domains (but not C2 alone) abrogated activation by Ubvs, demonstrating that Ubvs activate Ub transfer by relieving autoinhibitory interactions mediated by the C2 and WW domains. Previous studies demonstrated that Ub.wt and C2 binding sites overlap^41^ and this result suggests that the same may be true for WW domains. Unexpectedly, Ubv.P2.3 was still able to activate WWP1, even in the absence of both C2 and WW domains, suggesting that binding of the Ubv to the N‐lobe exosite not only relieves autoinhibition but also allosterically activates the enzyme. Allosteric activation by Ub binding was observed in other studies for an E2^19^ and RING E3 ligases,^21^ but the above result was the first demonstration that HECT E3 ligases can also be allosterically activated through the N‐lobe exosite.

Since the N‐lobe exosite was previously implicated in binding substrate‐bound Ub and thus promoting polyubiquitination, the Ubvs targeting NEDD4L and WWP1 were further investigated in polyubiquitination assays testing their effect on processive ubiquitination. Interestingly, while Ubv.NL.1 clearly inhibited processive ubiquitination by NEDD4L, Ubv.P2.3 targeting WWP1 had little effect, suggesting that binding of substrate‐bound Ub to the N‐lobe exosite is less important for processive ubiquitination in the case of WWP1 relative to NEDD4L. These results are consistent with the modulatory activity of Ubv.NL.1 observed in autoubiquitination assays, where Ubv.NL.1 simultaneously activated NEDD4L by relieving autoinhibition and inhibited its function by interfering with processive ubiquitination. Consistent with the effect of Ubv.NL.1 on processive ubiquitination of NEDD4L, a small molecule targeting the N‐lobe exosite of NEDD4 also inhibited processive ubiquitination.^40^ However, unlike Ubv.NL.1, the small molecule displayed no activating effect, suggesting that it blocks Ub binding without relieving NEDD4 autoinhibition. The activating effects observed with Ubvs targeting the N‐lobe exosite suggest that it may be possible to develop small molecules that bind differently within the N‐lobe exosite and act as activators.

Several Ubvs were further tested in cellular assays to confirm their ability to function inside cells. All of the tested Ubvs demonstrated cellular functions consistent with their in vitro properties (Table 2). Interestingly, despite the modulatory effect of Ubv.NL.1 on NEDD4L autoubiquitination observed in vitro, Ubv.NL1.1 expression had an overall activating effect on NEDD4L function in cells (Table 2). Additionally, all Ubvs generated against HECT3 ligases were pooled and screened for their effects on cell migration. This screen identified several Ubvs whose targets (HACE1, SMURF2, and WWP1/2) were known to be involved in cell migration and which affected cell migration in accordance to their in vitro properties (Table 2). The screen also recovered Ubv.NL.1 targeting NEDD4L as a strong inhibitor of cell migration, identifying NEDD4L as a novel regulator of this process (Table 2).

In summary, members of the HECT3 ligase family are amenable to targeting with Ubvs that are specific, biologically active and possess a range of effects on enzyme function (Figure 3I). In most cases, only one type of Ubv (inhibitor, activator or modulator) was obtained against a single HECT E3 ligase. However, additional phage selections with tailored Ubv libraries and using existing Ubvs to block unwanted interactions may produce Ubvs with different action modes. This tool kit of Ubvs should prove invaluable for exploring HECT E3 ligase biology and for facilitating structure‐guided design of small molecule inhibitors.

## 4 Inhibitors of SCF E3 ligases

**Figure 4:**
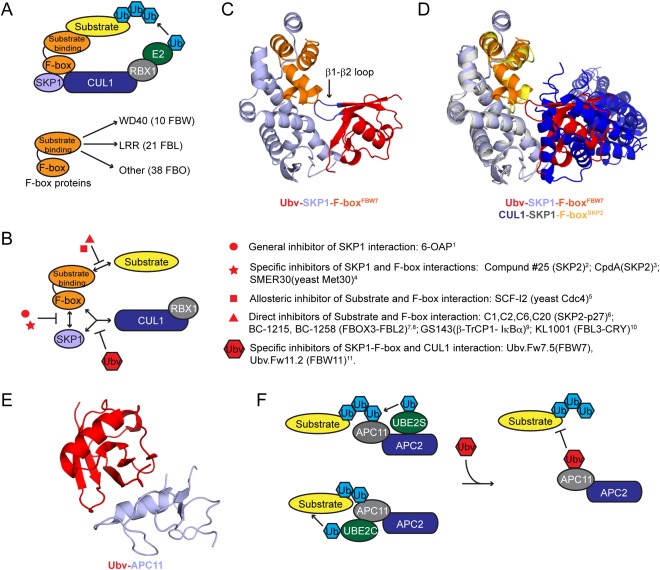
Ubv inhibitors of CRL E3 ligases. (A) Schematic representation of an SCF E3 ligase. (B) Examples of SCF E3 ligase inhibitors with diverse inhibition mechanisms. The references are as follows: 1,^46^ 2,^47^ 3,^48^ 4,^49^ 5,^50^ 6,^51^ 7,^52^ 8,^53^ 9,^54^ 10,^55^ 11.^56^ (C) Structure of Ubv.Fw7.1‐SKP1‐F‐box^FBW7^ complex (PDB: 5IBK). Ubv, SKP1, and F‐box^FBW7^ subunits are colored *red*, *light blue*, or *orange*, respectively. The Ubv β1‐β2 loop that interacts with the F‐box domain is labeled and colored blue. (D) Superposition of the Ubv.Fw7.1‐SKP1‐F‐box^FBW7^complex with the CUL1‐SKP1‐F‐box^SKP2^ (PDB: 1LDK) complex showing that Ubv.Fw7.1 and CUL1 interact with a similar surface. Subunits of the Ubv.Fw7.1‐SKP1‐F‐box^FBW7^ are colored as in (B) and CUL1‐SKP1‐F‐box^SKP2^ subunits are colored as follows: CUL1, *blue*; SKP1, *grey*; F‐box^SKP^ 2, *yellow*. (E) Structure of an Ubv‐APC11 complex (PDB: 5JG6). Ubv and APC11 are colored *red* or *light blue*, respectively. (F) Mechanism of inhibition by an Ubv targeting the APC11 Ub‐binding exosite. Binding of the substrate‐bound Ub to the APC11 exosite promotes multiubiquitination by UBE2C and chain elongation by UBE2S. Binding of an Ubv to the APC11 Ub‐binding exosite blocks binding of substrate‐bound Ub and interferes with ubiquitination

The SCF family of E3 ligases is one of the largest and best characterized of the CRL families (Figure 1). The SCF complex is composed of the invariant RBX1, CUL1, and SKP1 subunits, and one of the 69 F‐box proteins that determine substrate specificity (Figure 4A). The cullin protein CUL1 brings together the RING protein RBX1, which recruits the E2 enzyme, and the adaptor SKP1 in complex with the F‐box protein, which recruits substrate. All F‐box proteins are defined by the presence of a small F‐box domain that interacts with SKP1. The family is further subdivided according to the nature of the substrate‐binding domain including WD40, LRR and “other domains,” which are referred to as the FBW, FBL, and FBO subfamilies, respectively (Figure 4A). Numerous F‐box proteins control essential cell processes including cell cycle, DNA repair and apoptosis.^57^ Consequently, many F‐box proteins are attractive targets for treatment of cancer and other diseases. However, only a few F‐box proteins have been targeted with small molecules, with the majority of the effort devoted to SKP2, which drives cell cycle progression and is the best validated cancer target within the family (Figure 4B). Most reported inhibitors function by disrupting the interaction between an F‐box protein and its substrate. Additionally, inhibitors that disrupt the interaction of an F‐box domain with SKP1 were generated for SKP2 and the yeast protein Met30 (Figure 4B).

To gauge the potential of targeting F‐box proteins with Ubvs, we used the phage‐displayed Ubv library (Figure 2A) against the well‐characterized F‐box protein FBW7 in complex with SKP1.^56^ Unlike DUBs and HECT E3 ligases, Ub‐binding sites have not been characterized on most F‐box proteins, the exception being FBW family members which interact with Ub through the WD40 substrate‐binding domain that is thought to control auto‐ubiquitination.^58^ Unexpectedly, an Ubv (Fw7.1) generated against FBW7 in complex with SKP1 did not target its WD40 domain, but instead bound at the interface of the SKP1 protein and the F‐box domain (Figure 4C). Remarkably, the binding surface of Ubv.Fw7.1 overlapped almost exactly with the binding surface of CUL1 (Figure 4D), and consequently, the Ubv inhibited SCF^Fbw^ 7 activity by disrupting CUL1 binding. Thus, the Ubv‐phage screen identified a previously uncharacterized inhibitory site in the SCF E3 ligases defined by the interface of SKP1 and the F‐box domain. Since all members of the family contain an F‐box domain, in principle the whole F‐box family could be inhibited in a systematic manner by targeting this site.

To generate specific Ubv inhibitors against different F‐box proteins, we developed a next‐generation Ubv library tailored to target the SKP1‐F‐box interface. Characterization of the binding specificity of Ubv.Fw7.5 (affinity matured version of Ubv.Fw7.1) revealed that while it displayed the strongest binding to its target, it also bound to several related F‐box proteins in complex with SKP1 (Table 3). The observed cross‐reactivity was explained by the mechanism of Ubv binding (Figure 4C). The Ubv interaction with the F‐box domain, which confers specificity, is mediated exclusively by the β1‐β2 loop (Figure 4C), while the rest of the Ubv‐binding surface is in contact with SKP1. Based on this observation, a new library was made by randomizing and extending the β1‐β2 loop of Ubv.Fw.7.5 to increase the diversity of potential interactions with the F‐box domain while maintaining the favorable interactions with SKP1. This phage‐displayed library was used in binding selections against the F‐box protein FBW11 (β‐TrCP2). An Ubv generated against the SKP1‐F‐box^FBW11^ complex contained an eight‐residue insertion in the β1‐β2 loop and demonstrated remarkable specificity. It bound the closely related FBW1 (β‐Trcp1) protein very weakly and did not bind any of the other SKP1‐F‐box proteins tested (Table 3). The Ubvs generated against FBW7 and FBW11 were tested in cells and, consistent with their in vitro properties, disrupted the interactions of their cognate F‐box targets with CUL1 and inhibited degradation of known F‐box substrates (Table 3).

**Table 3:** Characteristics of Ubv inhibitors of CRL E3 ligases

Ubv (target)	Mutations^b^	Affinity^c^	Specificity^d^	Binding site	Biological activity	PDB code
Ubv.Fw7.1 (SKP1tr^a^‐FBW7)	17(12)	70 nM	Not determined	SKP1‐ F‐box interface		5IBK
Ubv.Fw7.5 (SKP1‐FBW7)	15	100 nM	FBW2 (0.76 μM) SKP2 (3.3 μM) FBW5 (>5 μM)	SKP1‐ F‐box interface	Disrupts interaction between CUL1 and FBW7 in cell lysates. Stabilizes FBW7 substrates c‐Myc and Cyclin E.	
Ubv.Fw11.2 (SKP1‐ FBW11)	21	130 nM	FBW1 (>5 µM)	SKP1‐ F‐box interface	Disrupts interaction between CUL1 and FBW11 in cell lysates. Stabilizes FBW11 substrates Cdc25 and Wee1.	
Ubv (APC11)	17(10)	1.6 µM	Specific	Ub‐binding exosite	Decreases APC/C dependent Cyclin B degradation in *Xenopus* egg extract system	5JG6

a SKP1tr denotes SKP1 with deletion of residues 38–43 and 70–81.

b The number of amino acid substitutions in Ubv relative to Ub.wt is shown. For Ubvs with solved complex structures the number of mutated residues that are located within the binding interface is indicated in parenthesis.

c For Ubv.Fw7.1, Ubv.Fw7.5, and Ubv.Fw11.2 affinities are represented by IC_50_ values calculated as the concentration of SKP1‐F‐box complex in solution that blocks 50% of Ubv binding to immobilized SKP1‐F‐box complex.^56^ The affinity of Ubv for APC11 was measured by Surface Plasmon Resonance (SPR).^59^

d For Ubv. Fw7.5 and Ubv.Fw11.2 specificity was determined by assessing binding to 6 different SKP1‐F‐box complexes. Cross‐reactive SKP1‐F‐box complexes and associated affinities are indicated. Specificity of Ubv targeting APC11 was assessed by testing binding to SCF^Fbw7^ and WWP1 HECT E3 ligases using in vitro substrate ubiquitination assays.

Ubvs generated against F‐box proteins show that members of this family can be inhibited in a systematic manner by targeting the CUL1‐binding surface on the SKP1‐F‐box complex and that highly specific Ubvs can be obtained. This inhibitory site was not previously discovered by small molecule studies and offers several advantages (Figure 4B). The whole F‐box family can be targeted in a systematic manner by this approach without prior knowledge of the F‐box‐substrate interactions, which are poorly characterized in most cases. Additionally, the SKP1‐F‐box domain complexes are easily purified and amenable to structural studies, thus facilitating the search for inhibitors. Interestingly, CUL1 was found to have extremely high affinity in vitro for the SKP1‐F‐box complex^56^, ^59^ and this may have prevented the identification of small molecules inhibitors that disrupt CUL1 binding by previous studies. However, despite very tight binding of CUL1 observed in vitro, Ubvs were still able to disrupt SKP1‐F‐box interaction with CUL1 in cells, most likely due to the action of exchange factor CAND1 that promotes dissociation of CUL1 in cells.^59^ This suggests that small molecule inhibitors of CUL1 binding could potentially be identified through in vitro assays that screen for the displacement of Ubvs from SKP1‐F‐box complexes. We anticipate that specific Ubv inhibitors targeting the SKP1‐F‐box interface can be generated against a significant proportion of the F‐box family and would provide valuable tools for validation of therapeutic targets and assist in development of inhibitory small molecules.

## 5 Inhibitors of APC/C

The APC/C complex contains at least 15 different core subunits and is the most elaborate of the CRL E3 ligases.^60^ The organization of the catalytic core resembles that of the SCF E3 ligases, as it contains the cullin subunit APC2, the RING protein APC11, the adaptor protein APC10, and two interchangeable substrate binding subunits CDC20 and CDH1. APC/C plays a central role during cell cycle, where APC/C^CDC20^ is responsible for driving the anaphase transition and mitotic exit, while APC/C^CDH1^ is mainly involved in governing transition through the G1 phase.^61^ Considering the central role of APC/C in cell cycle progression, it represents an attractive target for cancer therapy especially in the case of the APC/C^CDC20^ complex that is required for mitotic exit.^62^ To date, two small molecule inhibitors that either block CDC20 and CDH1 interaction with APC/C^63^ or disrupt substrate binding to CDC20^64^ have been generated, and they provide some insight into the mechanisms and outcomes of APC/C inhibition. However, given the central role of the APC/C complex in cell biology and its immense complexity, development of additional reagents would be highly beneficial for investigating APC/C function and assessing the consequences of targeting different sites.

Phage‐displayed libraries (Figure 2A) were used to generate Ubvs targeting APC11, the RING subunit of APC/C (Table 3).^65^ APC11 contains an Ub‐binding exosite, which presumably serves to capture substrate‐linked Ub in proximity to the E2 active site and contributes to chain elongation mediated by the UBE2S E2 enzyme.^66^ Analysis of the APC11‐Ubv complex coupled with NMR and enzyme assays demonstrated that the Ubv binds through the same interface and targets the same surface on APC11 as Ub.wt (Figure 4E). Accordingly, the Ubv impeded in vitro multiubiquitination mediated by the UBE2C E2 enzyme and chain elongation mediated by the UBE2S E2 enzyme in the same manner as the mutations to the APC11 Ub‐binding exosite (Figure 4F). The inhibitory effect of Ubv on APC/C function was also observed in a *Xenopus* egg system (Table 3).

The APC11‐binding Ubv was further used in cryo‐EM reconstructions to define architectures of Substrate‐Ub‐APC/C in complex with either UBE2C E2 enzyme involved in multiubiquitination or UBE2S E2 enzyme involved in chain elongation.^65^ The use of the high affinity Ubv in place of the low affinity Ub.wt was combined with cross‐linking of E2 enzymes to allow visualization of the APC/C complexes that are otherwise too transient to characterize structurally. Cryo‐EM structures and biochemical assays revealed that UBE2C and UBE2S E2 enzymes interact with the Substrate‐Ub‐APC/C complex in a strikingly different manner and provided an explanation for the non‐overlapping roles of the two enzymes in multiubiquitination and chain elongation.

The APC11‐binding Ubv provides yet another example of Ubvs mimicking the interaction of Ub.wt and demonstrates the utility of using high affinity Ubvs for structural studies. While the Ubv was used to help define the architectures of the APC/C complexes, it may also prove useful for exploring the biological consequences of inhibiting the APC11 Ub‐binding exosite. Furthermore, given the complexity of the APC/C complex, it would be interesting to explore whether Ubv inhibitors or modulators can be generated against other subunits of the APC/C complex.

## 6 Conclusions

We have summarized the development of Ubvs targeting members of three major UPS families including DUBs, HECT E3 ligases and CRL E3 ligases. Although Ubvs were generated against three functionally and structurally distinct families, characterization revealed several common principles underlying binding mechanisms and functions. First, in most cases, Ubvs targeting characterized Ub.wt binding sites bound in a very similar manner to Ub.wt, as observed for Ubvs against DUBs and HECT E3 N‐lobe exosites. This demonstrates that Ubvs can be used as high affinity substitutes for Ub.wt to assist in epitope mapping and structure determination, such as the studies investigating DUB‐Ub interactions and the APC/C complex structure. Second, Ubvs were generated not only against known Ub‐binding sites, but also against other protein interaction surfaces. This was observed for the Ubvs targeting the E2‐binding site in the HECT E3 ligases and those targeting the CUL1‐binding site in the SCF E3 ligases. While it remains to be demonstrated, it is likely that these sites are natural Ub‐binding sites that serve to regulate protein function. Therefore, Ubvs can be used to target novel sites for inhibition or modulation of activity and to uncover uncharacterized Ub.wt interaction sites. Finally, some Ubvs acted as activators rather than inhibitors. An Ubv targeting the OTUB1 enhanced its binding to the UbcH5b‐Ub complex and the Ubvs targeting the HECT N‐lobe exosite had an overall activating effect on their targets. An activating effect of Ub.wt binding to E2^19^ and E3 enzymes^21^ has been observed in several other studies suggesting that it should be possible to generate Ubv activators of other UPS proteins.

In conclusion, Ubvs developed against UPS proteins can serve as valuable tools both for basic research and for the development of therapeutic strategies. Several applications have been demonstrated in the described studies, including using high affinity Ubvs in place of Ub.wt for structural and functional studies, using Ubv pools to validate and discover new functions for targets, and using Ubvs to identify and characterize new modulatory sites. Other potential applications such as the use of Ubvs in small molecule displacement screens and therapeutic target validation remain to be explored.
